# Genotypic Variation in Grain P Loading across Diverse Rice Growing Environments and Implications for Field P Balances

**DOI:** 10.3389/fpls.2016.01435

**Published:** 2016-09-27

**Authors:** Elke Vandamme, Matthias Wissuwa, Terry Rose, Ibnou Dieng, Khady N. Drame, Mamadou Fofana, Kalimuthu Senthilkumar, Ramaiah Venuprasad, Demba Jallow, Zacharie Segda, Lalith Suriyagoda, Dinarathna Sirisena, Yoichiro Kato, Kazuki Saito

**Affiliations:** ^1^Africa Rice CenterDar es Salaam, Tanzania; ^2^Crop Production and Environment Division, Japan International Research Centre for Agricultural ScienceTsukuba, Japan; ^3^Southern Cross Plant Science, Southern Cross UniversityLismore, NSW, Australia; ^4^Southern Cross GeoScience, Southern Cross UniversityLismore, NSW, Australia; ^5^Africa Rice CenterBouaké, Côte d'Ivoire; ^6^Africa Rice CenterIbadan, Nigeria; ^7^National Agricultural Research InstituteBrikama, Gambia; ^8^Programme Riz et Riziculture, CNRST/INERABobo Dioulasso, Burkina Faso; ^9^Department of Crop Science, Faculty of Agriculture, University of PeradeniyaPeradeniya, Sri Lanka; ^10^Rice Research and Development InstituteIbbagamuwa, Sri Lanka; ^11^Crop and Environmental Sciences Division, International Rice Research InstituteMetro Manila, Philippines; ^12^Africa Rice CenterCotonou, Benin

**Keywords:** grain P concentration, P removal, P utilization efficiency, rice genotypes, P cycling

## Abstract

More than 60% of phosphorus (P) taken up by rice (*Oryza* spp.) is accumulated in the grains at harvest and hence exported from fields, leading to a continuous removal of P. If P removed from fields is not replaced by P inputs then soil P stocks decline, with consequences for subsequent crops. Breeding rice genotypes with a low concentration of P in the grains could be a strategy to reduce maintenance fertilizer needs and slow soil P depletion in low input systems. This study aimed to assess variation in grain P concentrations among rice genotypes across diverse environments and evaluate the implications for field P balances at various grain yield levels. Multi-location screening experiments were conducted at different sites across Africa and Asia and yield components and grain P concentrations were determined at harvest. Genotypic variation in grain P concentration was evaluated while considering differences in P supply and grain yield using cluster analysis to group environments and boundary line analysis to determine minimum grain P concentrations at various yield levels. Average grain P concentrations across genotypes varied almost 3-fold among environments, from 1.4 to 3.9 mg g^−1^. Minimum grain P concentrations associated with grain yields of 150, 300, and 500 g m^−2^ varied between 1.2 and 1.7, 1.3 and 1.8, and 1.7 and 2.2 mg g^−1^ among genotypes respectively. Two genotypes, Santhi Sufaid and DJ123, were identified as potential donors for breeding for low grain P concentration. Improvements in P balances that could be achieved by exploiting this genotypic variation are in the range of less than 0.10 g P m^−2^ (1 kg P ha^−1^) in low yielding systems, and 0.15–0.50 g P m^−2^ (1.5–5.0 kg P ha^−1^) in higher yielding systems. Improved crop management and alternative breeding approaches may be required to achieve larger reductions in grain P concentrations in rice.

## Introduction

Phosphorus (P) is a key nutrient limiting crop growth, and although it is needed by plants in lower total quantities than nitrogen (N) and potassium (K), its continued supply to crops is challenged by the finiteness of phosphate rock stocks worldwide. A large proportion of the P taken up by agricultural crops ends up in the food cycle without being recycled back to fields, leading to a continuous removal of P from fields (Smil, 2000; Senthilkumar et al., 2014; Wu et al., 2016). This results in high requirements for P inputs which come at a significant economic cost, and this cost is expected to increase in the future as high grade, readily accessible phosphate rock reserves are further depleted (Cordell et al., 2009; Senthilkumar et al., 2011, 2012). Where P removed from fields is not replaced by P-containing inputs, soil P stocks are gradually depleted, leading to soil degradation and a decline in productivity (Nziguheba et al., 2016). Highly negative P balances are commonly observed in agricultural fields in many developing countries (MacDonald et al., 2011; Fixen et al., 2015).

Improving the efficiency of P use in agriculture can be achieved by adapting agronomic management strategies to better exploit existing soil P stocks and new P inputs or by exploiting genotypic variation in P efficiency to breed more P-efficient crop cultivars (Simpson et al., 2011). Conventional P efficiency traits that have been targeted in crop improvement programs are P uptake efficiency (PAE, enhanced capacity of the plant to take up P from the soil) and P utilization efficiency (PUE, higher biomass production per unit of P taken up) (Wang et al., 2010; Rose and Wissuwa, 2012). In rice, 60–90% of P taken up by the crop is typically accumulated in the grains at maturity (Rose et al., 2010; Bi et al., 2013; Somaweera et al., 2015) and hence removed from the fields at harvest. Enhanced P uptake efficiency leads to higher yields but also to increased P removal from fields (Henry et al., 2010). Improved P utilization efficiency can either lead to higher grain yields (at equal levels of P uptake and P removal) or to reduced P removal from fields (at equal grain yield) (Vandamme et al., 2015). As grains contain the majority of P in the rice plant at maturity, and grains—including husks—are removed from fields, Rose et al. (2010) proposed to directly breed for low grain P concentrations as a way to reduce P removal from fields. A similar effort to lower P concentrations in maize (*Zea mays*) grains was undertaken by Wardyn and Russell (1998) with the aim of reducing environmental pollution associated with cattle manure. Recent studies on various crops including rice have focused on reducing phytate levels in grains because of human and animal health concerns, but total seed phosphorus generally remained unchanged (Dorsch et al., 2003; Bryant et al., 2005; Raboy, 2009). Some concerns have been raised about potential negative effects of reduced grain P concentration on seedling vigor. A number of studies have shown that such a negative response of seedling vigor to low grain P concentration can occur but is genotype-specific (Rose et al., 2012; Pariasca-Tanaka et al., 2015). Furthermore, the negative impact of low grain P concentration was shown to be small compared to genotypic variation in seedling vigor and plant responses to externally applied P. Breeding for reduced grain P concentrations is only feasible, however, if genotypic variation for this trait is sufficiently large to be exploited in breeding programs with a significant impact on removal of P from fields (Rose et al., 2013). In a field study in Japan, rice grain P concentration varied from 2.0 to 3.2 mg g^−1^ among 38 diverse rice genotypes (Rose et al., 2010), suggesting that considerable genotypic variation exists for this trait in rice. In a field study at three locations in Laos by Inthapanya et al. (2000), grain P concentrations of 16 rice genotypes were lower (on average 1.6 mg g^−1^) but significantly affected by a location × P rate × genotype interaction. The concentration of P in rice grains is determined by a complex interplay between P supply and other grain yield-determining factors, which can be environmental or genotypic in origin or affected by the interaction of both (Vandamme et al., 2015). In order to identify donor genotypes with a low grain P concentration and understand the implications of genotypic variation in grain P concentration for field P balances, it is essential to take into account this interplay by determining grain P concentration of rice genotypes grown in a wide range of environments with and without external P supply. This study therefore aimed to: (i) assess genotypic variation in grain P concentration of rice in a diverse range of rice growing environments, (ii) identify genotypes with a low grain P concentration irrespective of grain yield, and (iii) quantify the potential impact of the observed genotypic variation in grain P concentration on P removal from rice fields. For these purposes, a series of multi-location experiments was established at different sites across Africa and Asia within the framework of the Global Rice Science Partnership (GRiSP, www.grisp.net).

## Materials and methods

### Theoretical framework

**Figure 1:**
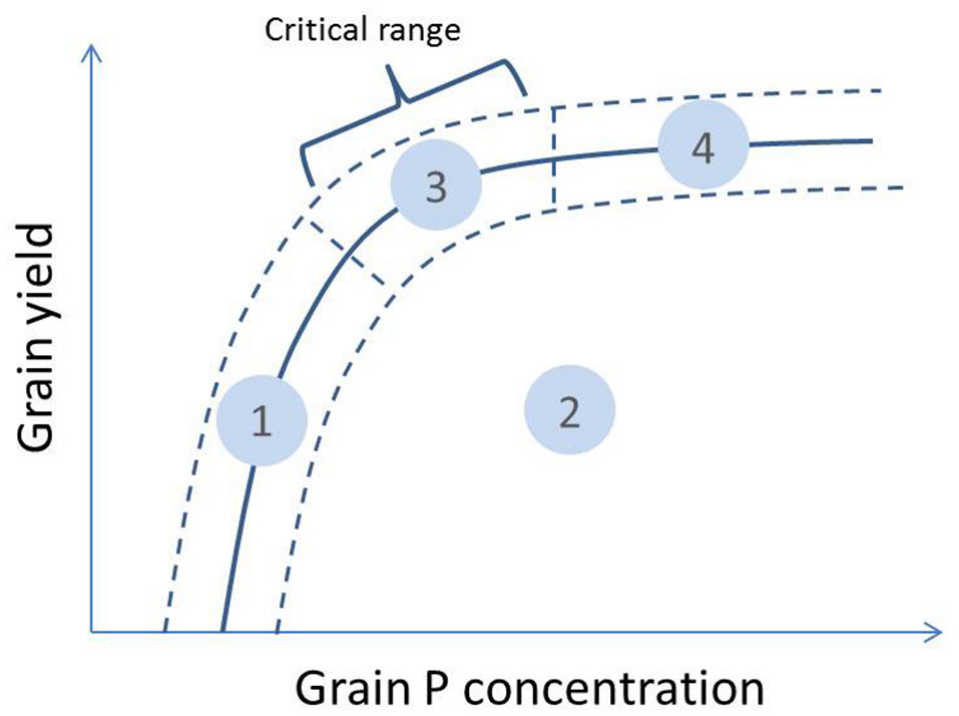
**Theoretical relationship between grain yield and grain P concentration of a genotype grown across different environments or levels of P supply (full line with uncertainty interval indicated with dashed lines)**. The different zones on the curve can be interpreted as: (1) low grain yield and low grain P concentration—grain yield restricted by P availability; (2) low grain yield and medium to high grain P concentration—grain yield restricted by other factors; (3) high grain yield and medium grain P concentration—no major restrictions to grain yield; (4) high grain yield and luxury grain P loading—no major restrictions to grain yield and very high P supply. The critical range is the portion of the curve where yield declines quickly with declining grain P concentration.

A typical nutrient response curve is characterized by an ascending area of the curve where yield increases with plant nutrient concentration, and a relatively level portion where yield is not limited by the specific nutrient (Bates, 1971). The portion of the curve where yield declines quickly with declining nutrient concentration is referred to as the “critical range.” Based on this, a theoretical relationship between grain yield and grain P concentration for a certain rice genotype grown across different environments or levels of P supply was drawn in Figure 1. In P-deficient environments, grain yield is limited by insufficient P uptake and this affects grain P concentrations which remain low (zone 1 on Figure 1). When P supply increases, increases in grain P concentration will depend on whether grain yield increases concomitantly. If other factors limiting grain yield exist, additional P available in the plant tissue will be distributed among a small amount of grain biomass and grain P concentrations may ultimately reach high levels (zone 2). In environments where P deficiency is the main yield-limiting factor, additional P uptake will lead to increases in grain P concentration and grain yield but—compared to zone 2—the additional P moving to grain may be partly diluted because of the grain yield increase (zone 3). Lastly, if P supply is in excess of that required by the crop for optimal growth, luxury P loading in grains at concentrations above the sufficiency range may occur (zone 4).

When comparing genotypes within the same environment, both a positive and negative relationship between grain P concentrations and grain yields can exist among genotypes. A positive relationship can occur when genotypes with a higher capacity to acquire P have a larger amount of P available for growth and consequently a higher P concentration in their biomass and also grains. A negative trend may occur when higher grain yield leads to lower grain P concentration due to a “dilution effect” (Batten, 1992; McDonald et al., 2008) or when higher grain yield is the result of superior P utilization efficiency. When applied to environmental variation, the positive and negative relationships between grain yield and grain P concentration translates into shifts from zone 1 to 3, and 2 to 3 in Figure 1, respectively.

### Multi-location genotype screening experiments

Twenty three field trials were conducted between 2012 and 2014 using 10–75 genotypes per trial (Table 1). In total, 83 different genotypes were tested across locations. The selection of genotypes was based on initial data on grain P concentrations (Wissuwa et al., 2015), their previous performance under P deficiency (Mori et al., 2016) and on general adaptation to conditions at respective sites. In all the trials, with the exception of trials 3, 19, 20, 21, and 23 (see trial numbers in Table 1), genotypes were grown at two P rates (with and without P application) in different but neighboring field plots. In trials 3 and 19, genotypes were grown only with P application and in trials 20, 21, and 23, genotypes were grown only without P application. Herein, each trial × P rate combination was considered as one environment and data were collected in 41 environments in total.

**Table 1:** **List of trial and environment numbers with information on year, country, site, rice growing environment, P treatment and number of genotypes, and probabilities of F-statistics for single-environment ANOVA for the effect of genotype on grain yield, grain P concentration, straw biomass and straw P concentration in each environment**.

**Env**	**Trial**	**Year**	**Country**	**Site**	**Rice growing environment**	**P treatment**	**No. of genotypes**	**Grain yield**	**Grain P conc**	**Straw biomass**	**Straw P conc**
1	1	2012	Benin	Bohicon	Upland	+	39	^**^	ns	nd	nd
2	1	2012	Benin	Bohicon	Upland	−	39	^**^	ns	nd	nd
3	2	2012	Burkina Faso	Farako-ba	Upland	+	39	^***^	ns	nd	nd
4	3	2012	The Gambia	Yundum	Upland	+	39	^*^	ns	nd	nd
5	3	2012	The Gambia	Yundum	Upland	−	39	^*^	ns	nd	nd
6	4	2012	Benin	Cotonou	Upland	+	40	^***^	^**^	^***^	^***^
7	4	2012	Benin	Cotonou	Upland	−	40	^***^	^***^	^***^	^***^
8	5	2012	Benin	Cotonou	Lowland	+	75	^***^	^*^	^***^	^***^
9	5	2012	Benin	Cotonou	Lowland	−	75	^***^	^***^	^***^	^***^
10	6	2012	Philippines	Pangil	Lowland	+	19	^**^	^***^	^***^	^***^
11	6	2012	Philippines	Pangil	Lowland	−	19	ns	^*^	^***^	^*^
12	7	2013	Nigeria	Ibadan	Lowland	+	50	^***^	^**^	^***^	^***^
13	7	2013	Nigeria	Ibadan	Lowland	−	50	^***^	^**^	^***^	^***^
14	8	2013	Benin	Cotonou	Lowland	+	21	ns	^***^	^*^	nd
15	8	2013	Benin	Cotonou	Lowland	−	21	^***^	^***^	ns	nd
16	9	2013	Benin	Cotonou	Upland	+	12	ns	^**^	^***^	ns
17	9	2013	Benin	Cotonou	Upland	−	12	^**^	^*^	^**^	^*^
18	10	2013	Benin	Cotonou	Upland	+	12	^*^	^*^	^***^	^**^
19	10	2013	Benin	Cotonou	Upland	−	12	ns	^**^	ns	^*^
20	11	2013	Benin	Cotonou	Upland	+	12	^**^	^*^	^**^	ns
21	11	2013	Benin	Cotonou	Upland	−	12	^*^	^*^	^**^	^*^
22	12	2013	Benin	Bohicon	Upland	+	33	^***^	^***^	^***^	^***^
23	12	2013	Benin	Bohicon	Upland	−	33	^***^	^***^	^***^	^***^
24	13	2013	Burkina Faso	Farako-ba	Upland	+	33	ns	0.05	ns	ns
25	13	2013	Burkina Faso	Farako-ba	Upland	−	33	^*^	^*^	ns	^*^
26	14	2013	Nigeria	Ikenne	Upland	+	33	^**^	^**^	^***^	^***^
27	14	2013	Nigeria	Ikenne	Upland	−	33	^***^	^*^	^***^	^**^
28	15	2013	Benin	Cotonou	Upland	+	33	^***^	ns	^**^	^**^
29	15	2013	Benin	Cotonou	Upland	−	33	^***^	0.06	^**^	^**^
30	16	2013	Tanzania	Dakawa	Lowland	+	45	^***^	^*^	^**^	ns
31	16	2013	Tanzania	Dakawa	Lowland	−	45	^***^	^**^	ns	ns
32	17	2013	Tanzania	Ruvu	Lowland	+	43	^***^	^**^	^***^	^***^
33	17	2013	Tanzania	Ruvu	Lowland	−	43	^***^	0.07	^***^	ns
34	18	2013	Japan	Tsukuba	Upland	+	13	ns	^***^	ns	^***^
35	19	2013	Japan	Tsukuba	Upland	−	13	ns	^***^	ns	ns
36	20	2013	Japan	Tsukuba	Upland	−	13	^*^	ns	^*^	ns
37	21	2013	Philippines	Pangil	Lowland	+	18	^*^	^***^	^***^	^***^
38	21	2013	Philippines	Pangil	Lowland	−	18	^***^	^**^	^***^	^**^
39	22	2014	Sri Lanka	Bathalagoda	Lowland	−	20	^***^	^***^	nd	^***^
40	23	2014	Tanzania	Dakawa	Lowland	+	10	^***^	^***^	^*^	^**^
41	23	2014	Tanzania	Dakawa	Lowland	−	10	^***^	ns	^**^	ns

*** *P < 0.001*,

** *P < 0.01*,

* *P < 0.05*,

*ns, not significant; nd, not determined*.

Detailed information on soil characteristics, fertilizer rates, experimental design, plot size, hill density and number of replicates is provided in Supplementary Table 1. A full list of all genotypes evaluated can be found in Supplementary Datasheet 1. Fourteen trials were conducted in West Africa, three in East Africa and six in Asia. Nine trials were conducted under irrigated lowland conditions (flooded) while 14 trials were conducted under upland (aerobic) conditions. Under upland conditions, rainfall was supplemented with irrigation if needed except for the trials in Burkina Faso and The Gambia which were strictly rainfed. The trials were established with three replicates in each environment, with the exception of trial 20 which was conducted with 2 replicates. The pH (1:5 H_2_O) of the soils ranged from 4.7 to 6.9 and soil P availability (Bray-P) ranged between 1.3 and 18 mg P kg^−1^. An alpha lattice design was used in 14 trials and a randomized complete block design in the other nine trials. Trials in upland environments were established by direct seeding (dibbling) and trials in lowland were established by transplanting seedlings that were raised in a nursery bed. Plot size ranged from 0.4 to 3 m^2^ depending on the trial. Nitrogen and potassium (K) were applied in all the trials at rates ranging from 75 to 150 kg N ha^−1^ and 30 to 50 kg K ha^−1^, respectively. Where P was applied, its rates ranged from 22 to 30 kg P ha^−1^.

At harvest, aboveground biomass was collected, separated into panicles and straw, and grains were manually threshed. Straw was oven-dried at 65°C until constant weight while filled grains were air-dried, weighed and their grain moisture content determined. Grain yields are presented at 14% moisture content. Grain and straw samples were ground and digested following different digestion protocols. In Africa, the samples were digested in sulfuric acid, salicylic acid, hydrogen peroxide, and selenium (Novozamsky et al., 1983) and plant P concentration was determined by colorimetry using a continuous-flow analysis system (Thomas et al., 1967). Samples from Asia were digested in a mixture of 3:1:1 nitric:perchloric:sulfuric acid and the P concentration in the extract was determined using the colorimetric vanadomolybdate assay (Murphy and Riley, 1962). Data on grain yield and grain P concentration for all 41 environments were compiled. For 34 and 35 out of these 41 environments, data on straw biomass and straw P concentration were also obtained, respectively.

### Data analysis

Firstly, single-environment ANOVAs were carried out to evaluate differences in grain yield, straw biomass, grain P concentration and straw P concentration among genotypes within each environment and to calculate least square means for these variables in each environment using SAS software (SAS Institute Inc., 2012). A mixed model (PROC MIXED) was used with genotype as a fixed factor and replicate and block nested into replicate (in the case of alpha lattice design) as random factors.

For the second part of the analysis, 30 genotypes were selected that had been grown in at least 15 out of the 41 environments. These genotypes, and information on their origin, species group and the number of environments in which they were grown, are presented in Table 2. The minimum and maximum number of genotypes taken into account per environment after this selection was 7 and 30, respectively, with an average of 20 (Table 3). A cluster analysis was then carried out with the aim to group environments based on average grain yield and grain P concentration in each environment following the theoretical framework presented in Figure 1. The method of cluster analysis used was hierarchical complete-linkage clustering based on Euclidian distance using the R software version 3.3.0 (R Core Team, 2016). Subsequently, genotypic variation in grain yield, grain P concentration and straw P concentration was evaluated within each of the environment clusters. To avoid bias in genotype means across environments due to the unbalanced design of the multi-location trials (not all genotypes grown in all environments), standard scores of the outcome variables (grain yield, grain P concentration and straw P concentration) for each of the genotypes within each environment were calculated as follows:

(1) $$\begin{array}{l} Yi\left(j\right),std=\frac{Yi\left(j\right)-Yj}{\sigma j} \end{array}$$

where *Yi(j), std* is the standard score for variable *Y* of genotype *i* within environment *j, Yi(j)* is the observed value for variable *Y* of genotype *i* within environment *j*, and *Yj* and σ*j* are the mean and standard deviation among genotypes for variable *Y* in environment *j*. Average standard scores per environment cluster were calculated for each genotype and compared among genotypes within each environment cluster by a mixed model analysis in SAS with genotypes as fixed factor and environment as random factor, and standard errors of the differences were calculated.

**Table 2:** **Genotypes selected for cluster analysis with information on country of origin, genetic group and number of environments in which they were grown**.

**Genotype**	**Country of origin**	**Group**	**#Env**
Apo	Philippines	IND	15
BJ1	India	IND (AUS)	30
Coarse	Pakistan	IND (AUS)	23
Dawebyan	Myanmar	IND	31
DJ123	Bangladesh	IND (AUS)	39
EMATA A16-34	Myanmar	IND	22
IR36	Philippines	IND	33
IR64	Philippines	IND	32
IR8	Philippines	IND	21
IR82635-B-B-143-1	Philippines	IND	25
IR82635-B-B-93-2	Philippines	IND	17
IR83399-B-B-52-1	Philippines	IND	19
ITA257	Nigeria	TRJ	31
Kalubala Vee	Sri Lanka	IND (AUS)	34
Kasalath	India	IND (AUS)	23
Mudgo	India	IND	38
NERICA1	Ivory Coast	Interspecific	25
NERICA10	Ivory Coast	Interspecific	23
NERICA3	Ivory Coast	Interspecific	21
NERICA4	Ivory Coast	Interspecific	30
PH218-5-3-8-3	Philippines	IND	19
Sadri Tor Misri	Iran	ADMIX	39
Santhi Sufaid	Pakistan	IND (AUS)	41
Seratous Heri	Indonesia	IND	15
Sigadis	Indonesia	IND	17
Surjamkuhi	India	IND (AUS)	38
Taichung Native1	Taiwan	IND	30
Tondok	Indonesia	TRJ	16
TOX1011-4-A2	Nigeria	TRJ	31
Yodanya	Myanmar	IND	28

*ADMIX, admixture; IND, Indica; Interspecific, O. sativa × O. glaberrima; TRJ, tropical japonica; AUS, variety group from India/Bangladesh known for earliness and tolerance to stresses*.

**Table 3:** **Mean grain yield, straw biomass and grain and straw P concentration in each environment (across selected genotypes) and the number of the environment cluster in which they were grouped by cluster analysis based on grain yield and grain P concentration**.

**Env**	**Year**	**Country**	**Site**	**Ecology**	**P treatment**	**No. of selected genotypes**	**Grain yield (g m^−2^)**	**Straw biomass (g m^−2^)**	**Grain P conc (mg g^−1^)**	**Straw P conc (mg g^−1^)**	**Env cluster**
2	2012	Benin	Bohicon	Upland	−	24	287	nd	2.09	nd	1
3	2012	Burkina Faso	Farako-ba	Upland	+	24	237	nd	2.46	nd	1
5	2012	The Gambia	Yundum	Upland	−	22	349	nd	1.49	nd	1
11	2012	Philippines	Pangil	Lowland	−	16	82	112	1.77	0.50	1
19	2013	Benin	Cotonou	Upland	−	12	325	864	1.99	0.54	1
20	2013	Benin	Cotonou	Upland	+	12	241	658	1.83	0.32	1
21	2013	Benin	Cotonou	Upland	−	12	86	103	1.61	0.46	1
23	2013	Benin	Bohicon	Upland	−	25	133	168	2.19	0.50	1
25	2013	Burkina Faso	Farako-ba	Upland	−	26	75	80	1.58	0.48	1
27	2013	Nigeria	Ikenne	Upland	−	25	219	362	2.17	0.44	1
34	2013	Japan	Tsukuba	Upland	+	13	281	785	2.13	0.78	1
36	2013	Japan	Tsukuba	Upland	−	13	280	449	1.83	0.36	1
37	2013	Philippines	Pangil	Lowland	+	15	247	254	2.49	1.18	1
38	2013	Philippines	Pangil	Lowland	−	15	165	154	1.36	0.31	1
39	2014	Sri Lanka	Bathalagoda	Lowland	−	16	270	nd	1.57	0.22	1
41	2014	Tanzania	Dakawa	Lowland	−	7	322	289	2.12	0.81	1
6	2012	Benin	Cotonou	Upland	+	23	276	483	3.40	1.13	2
7	2012	Benin	Cotonou	Upland	−	23	268	420	3.23	1.42	2
8	2012	Benin	Cotonou	Lowland	+	30	323	421	2.85	1.37	2
10	2012	Philippines	Pangil	Lowland	+	16	115	156	3.31	1.74	2
12	2013	Nigeria	Ibadan	Lowland	+	23	249	338	3.21	0.81	2
13	2013	Nigeria	Ibadan	Lowland	−	22	299	358	3.20	1.01	2
14	2013	Benin	Cotonou	Lowland	+	19	254	332	3.53	1.53	2
22	2013	Benin	Bohicon	Upland	+	25	213	289	2.93	1.41	2
24	2013	Burkina Faso	Farako-ba	Upland	+	26	114	106	2.91	1.50	2
26	2013	Nigeria	Ikenne	Upland	+	24	319	474	3.06	1.03	2
29	2013	Benin	Cotonou	Upland	−	24	291	469	2.89	1.27	2
1	2012	Benin	Bohicon	Upland	+	24	363	nd	2.80	nd	3
4	2012	The Gambia	Yundum	Upland	+	22	402	nd	2.49	nd	3
9	2012	Benin	Cotonou	Lowland	−	30	444	474	3.11	1.23	3
16	2013	Benin	Cotonou	Upland	+	12	372	764	2.50	1.28	3
17	2013	Benin	Cotonou	Upland	−	12	414	675	2.13	1.12	3
18	2013	Benin	Cotonou	Upland	+	12	404	474	2.23	0.60	3
28	2013	Benin	Cotonou	Upland	+	26	357	534	2.83	1.25	3
30	2013	Tanzania	Dakawa	Lowland	+	24	555	900	2.70	0.92	3
31	2013	Tanzania	Dakawa	Lowland	−	25	469	782	2.86	0.87	3
35	2013	Japan	Tsukuba	Upland	−	13	367	682	2.45	0.67	3
40	2014	Tanzania	Dakawa	Lowland	+	8	538	549	2.26	1.11	3
15	2013	Benin	Cotonou	Lowland	−	19	452	428	3.90	1.43	4
32	2013	Tanzania	Ruvu	Lowland	+	25	493	715	3.61	1.62	4
33	2013	Tanzania	Ruvu	Lowland	−	25	368	660	3.66	1.54	4

The third part of the analysis was carried out using 14 genotypes that had been grown in at least 30 out of 41 environments. For each genotype, a response curve as illustrated in Figure 1 was plotted using the observed values for the genotypes in each of the environments. To evaluate the maximum attainable yield at a range of grain P concentrations, boundary curves were then fitted using the method described by Shatar and McBratney (2004). First, outliers were selected and removed for the boundary curve analysis. Two types of outliers were distinguished: (1) data points with yields more than 100 g m^−2^ higher than other yield levels of data points within a range of ±0.4 mg g^−1^ in grain P concentration, and (2) data points with the lowest grain P concentration observed among environments for a particular genotype yet with a grain yield level higher than 300 g m^−2^. The first type of outliers were removed to avoid bias in the upper part of the curve (maximum yield level) while the second type of outliers were removed to avoid bias in the lower and left part of the curves. Subsequently, quadratic spline boundary curves (Daouia et al., 2015) were fitted. For each of the genotypes, maximum grain yield and minimum grain P concentrations at various yield levels were then derived from the boundary curves, as well as minimum P removal at various grain yield levels. The minimum grain P concentrations were compared with average observed grain P concentrations in different grain yield level intervals for each genotype.

## Results

### Environmental variation and genotypic variation within single environments

Probabilities of F-statistics for the effect of genotype on grain yield, grain P concentration, straw biomass and straw P concentration within each environment are presented in Table 1. Significant differences (*P* < 0.05) in grain yield among genotypes were found in 85% of the environments, and for straw biomass in 80% of the environments. For grain and straw P concentration, significant differences were found in 73 and 74% of the environments, respectively. Differences among genotypes in these variables were equally detected in both environments with and without P applied. Least square means for grain yield, straw biomass, grain P concentration and straw P concentration in all the environments are presented in Supplementary Datasheet 1.

**Figure 2:**
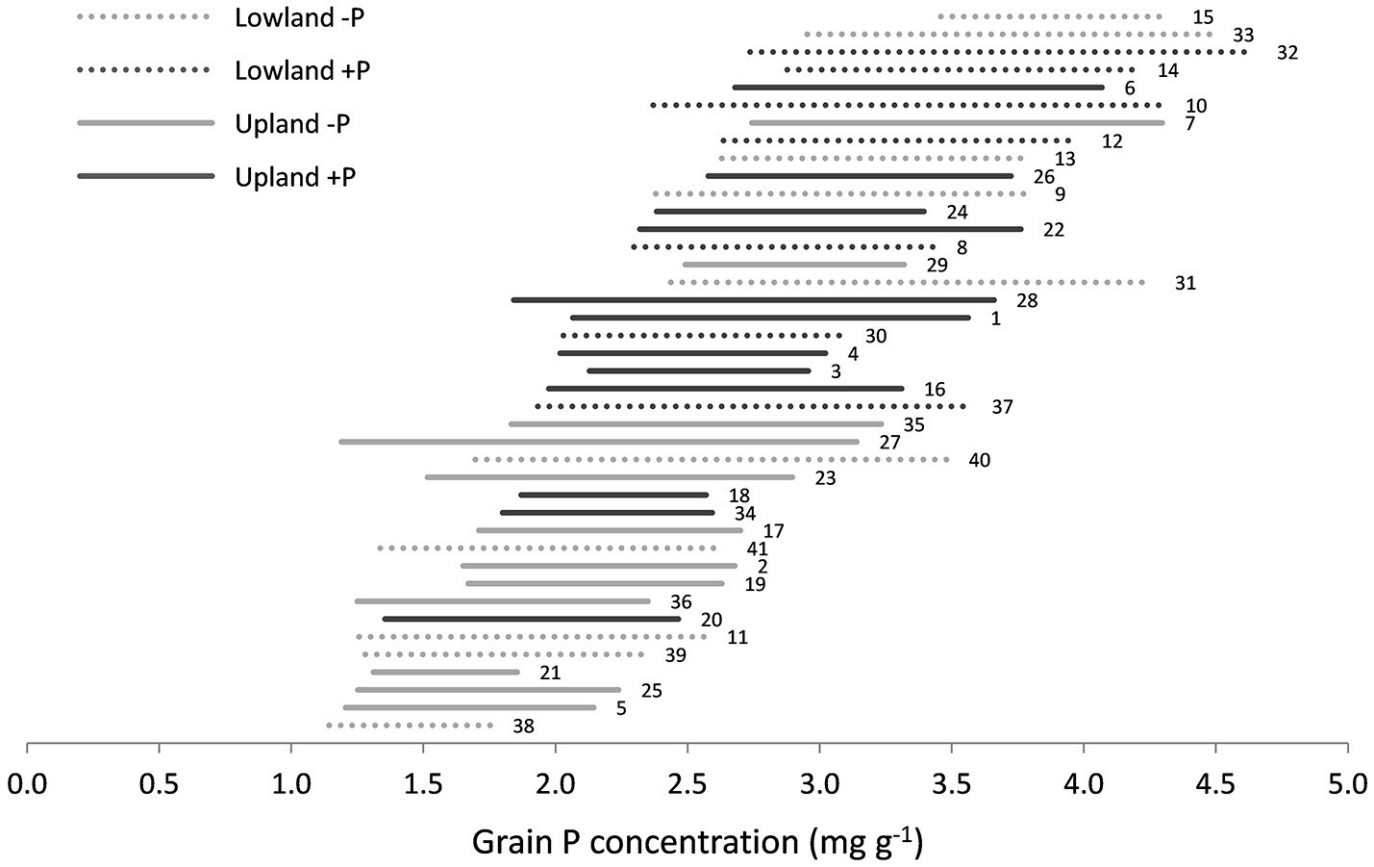
**Range in grain P concentration among genotypes in each environment with each line representing the minimum and maximum grain P concentration observed in a certain environment**. Environments are sorted from lowest (down) to highest (up) mean grain P concentration. The labels next to the lines are environment numbers as presented in Table 1.

Average grain P concentrations across genotypes varied almost 3-fold among environments, from 1.4 to 3.9 mg g^−1^ (Supplementary Datasheet 1). On average across environments, grain P concentration tended to be higher under lowland conditions than under upland conditions (2.8 vs. 2.4 mg g^−1^) and higher when P was applied (2.8 vs. 2.3 mg g^−1^). Within environments, grain P concentration varied 1.3- to 2.7-fold among genotypes (Supplementary Datasheet 1 and Figure 2). The difference between the minimum and maximum observed grain P concentration within one environment was on average 1.2 mg g^−1^ and ranged between 0.5 and 2.0 mg g^−1^ (Figure 2). The lowest observed grain P concentration was 1.1 mg g^−1^ for the genotype Dawebyan in environment 38 and the highest observed grain P concentration was 4.7 mg g^−1^ for the genotype Kalubala Vee in environment 32 (Supplementary Datasheet 1).

Average straw P concentrations across genotypes varied widely among environments, between 0.2 and 1.7 mg g^−1^ (Supplementary Datasheet 1). Within environments, straw P concentration varied between 1.8- and 7.5-fold among genotypes. The lowest observed straw P concentration was 0.1 mg g^−1^ for the genotype Kalubala Vee in environment 39 and the highest observed straw P concentration was 3.3 mg g^−1^ for the genotype PH228-2 in environment 22.

### Genotypic variation within environment clusters

**Figure 3:**
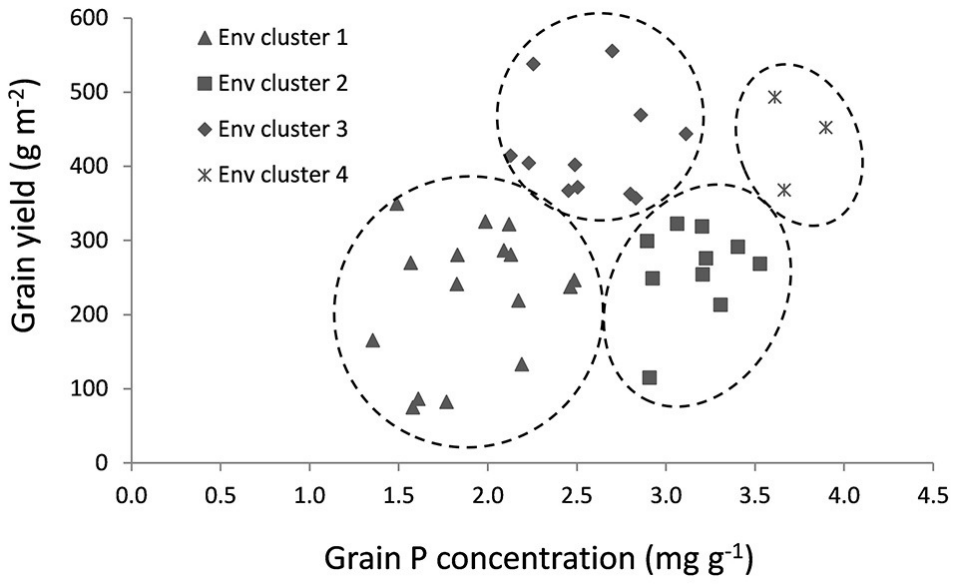
**Environments clustered based on mean grain yield and grain P concentration per environment**.

A cluster analysis to group environments based on mean grain yield and grain P concentration was carried out using data of a selection of 30 genotypes (Table 2) and the environments were grouped in four environment clusters. Mean grain yield, straw biomass, grain and straw P concentration per environment and the cluster in which they were grouped are shown in Table 3. Figure 3 visualizes the variation in grain yield and P concentration within and among the clusters, and mean grain yield, grain P concentration and straw P concentration per cluster are shown in Table 4. Environment clusters 1, 2, 3, and 4 comprised 16, 11, 11, and 3 environments, respectively. The first group (environment cluster 1) had relatively low grain yield and low grain P concentration, indicating that grain yield was restricted by P (P-limited environments). The second group (environment cluster 2) was characterized by relatively low grain yield but high grain P concentration. This indicated that a factor other than P was limiting grain yield. Environment cluster 3 was high-yielding with grain P concentrations around 2.5 mg g^−1^, which can be considered typical for rice (Dobermann et al., 1998), and was identified as the environment group in which there were no major yield-limiting factors. The last environment group had high grain yields with very high grain P concentrations indicating that excessive P supply led to luxury P uptake in the plants. Twelve out of 21 environments where no P was applied were grouped in environment cluster 1. Environment cluster 4 (luxury P supply) only contained lowland environments, while other clusters contained both upland and lowland environments.

**Table 4:**
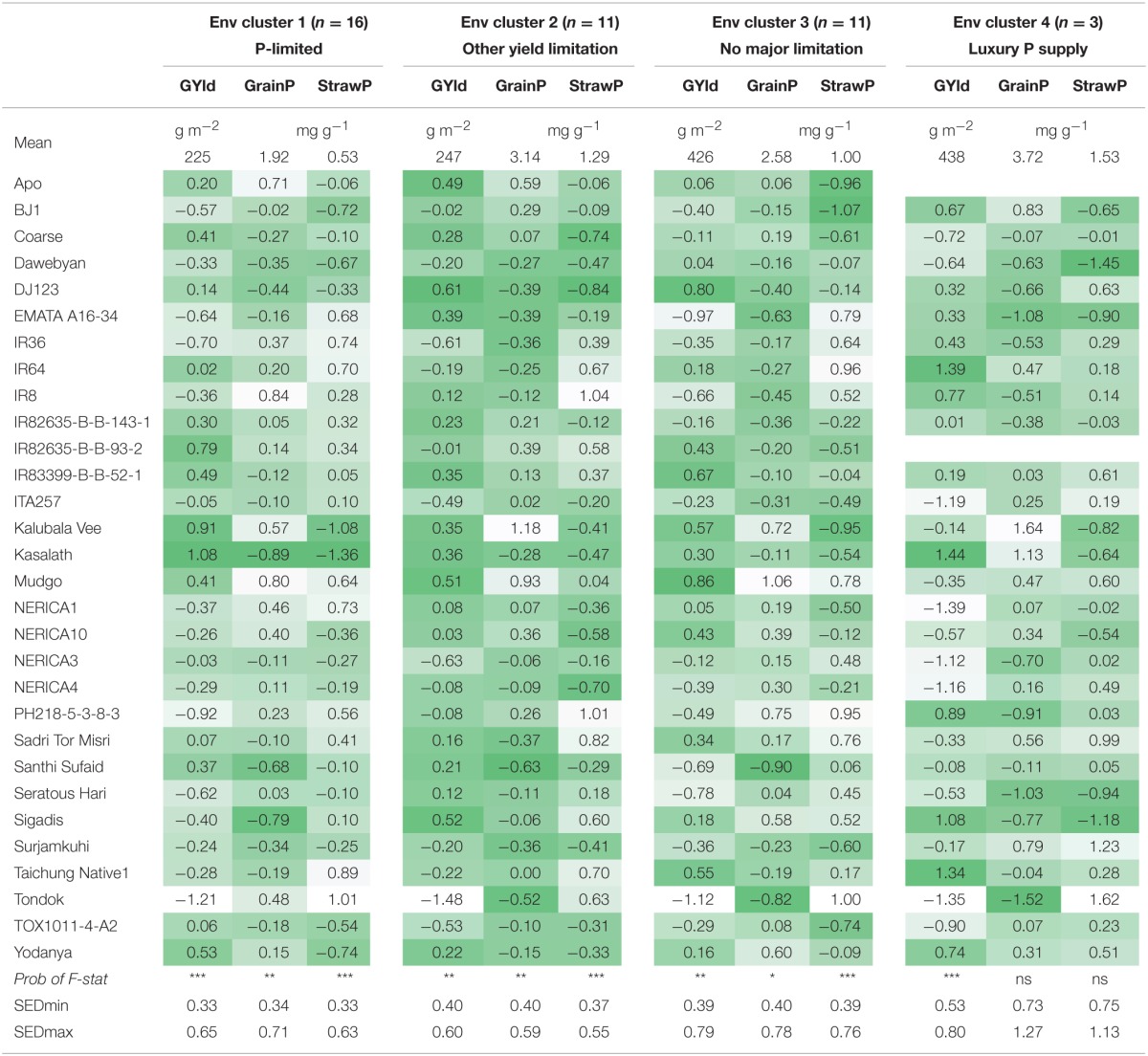
**Mean per cluster and standard scores per genotype for grain yield (GYld), grain P concentration (GrainP) and straw P concentration (StrawP) for 30 genotypes in different environment clusters**.

Environments were clustered based on mean grain yield and grain P concentration. SED is standard error of the difference; *** P < 0.001, ** P < 0.01, * P < 0.05, ns, not significant; for grain yield: dark color = high yield (desirable) to light color = low yield, for grain and straw P concentration: dark color = low P concentration (desirable) to light color = high P concentration.

In environment cluster 1 (P-limited environments), the genotype Kasalath had the highest grain yield combined with the lowest grain and straw P concentration (Table 4). To the contrary, Sigadis had low grain P concentration combined with low grain yields. Santhi Sufaid had considerably lower than average grain P concentration and moderately higher than average grain yield. Kalubala Vee and Mudgo had high grain yields and high grain P concentrations indicating superior P uptake.

In environment cluster 2 (grain yield restricted by other factors), Santhi Sufaid was the genotype with the lowest grain P concentration while its yield was moderately higher than average. Again, Kalubala Vee and Mudgo had high grain P concentrations associated with high grain yields.

In environment cluster 3 (no major yield-limiting factor), Santhi Sufaid again had low grain P concentrations but also lower than average grain yield. Tondok and EMATA A16-34 also had low grain P concentrations but this was associated with considerably lower than average grain yields. DJ123 had considerably lower grain P concentration while its grain yield was higher than average. As in environment cluster 1 and 2, Mudgo and Kalubala Vee had high grain yields and high grain P concentrations. Data from environment cluster 4 (luxury P supply) have to be interpreted with care as only three environments were grouped in this cluster. Results tended to be similar as those for environment cluster 3 but variation in grain and straw P concentration among genotypes was not significant in this cluster.

Santhi Sufaid was the only genotype that had the lowest grain P concentration in two environment clusters (cluster 2 and 3) and it was also among the three genotypes with lowest grain P concentration in environment cluster 1. DJ123 was the only genotype that had lower than average grain P concentration in all environment clusters combined with medium to high grain yield levels. Kalubala Vee and Mudgo had high grain P concentrations across all environment clusters, with Mudgo also having higher than average straw P concentrations while Kalubala Vee had considerably lower than average straw P concentrations indicating that its high grain P concentrations were associated with enhanced P translocation from the straw.

### Boundary curve analysis

**Figure 4:**
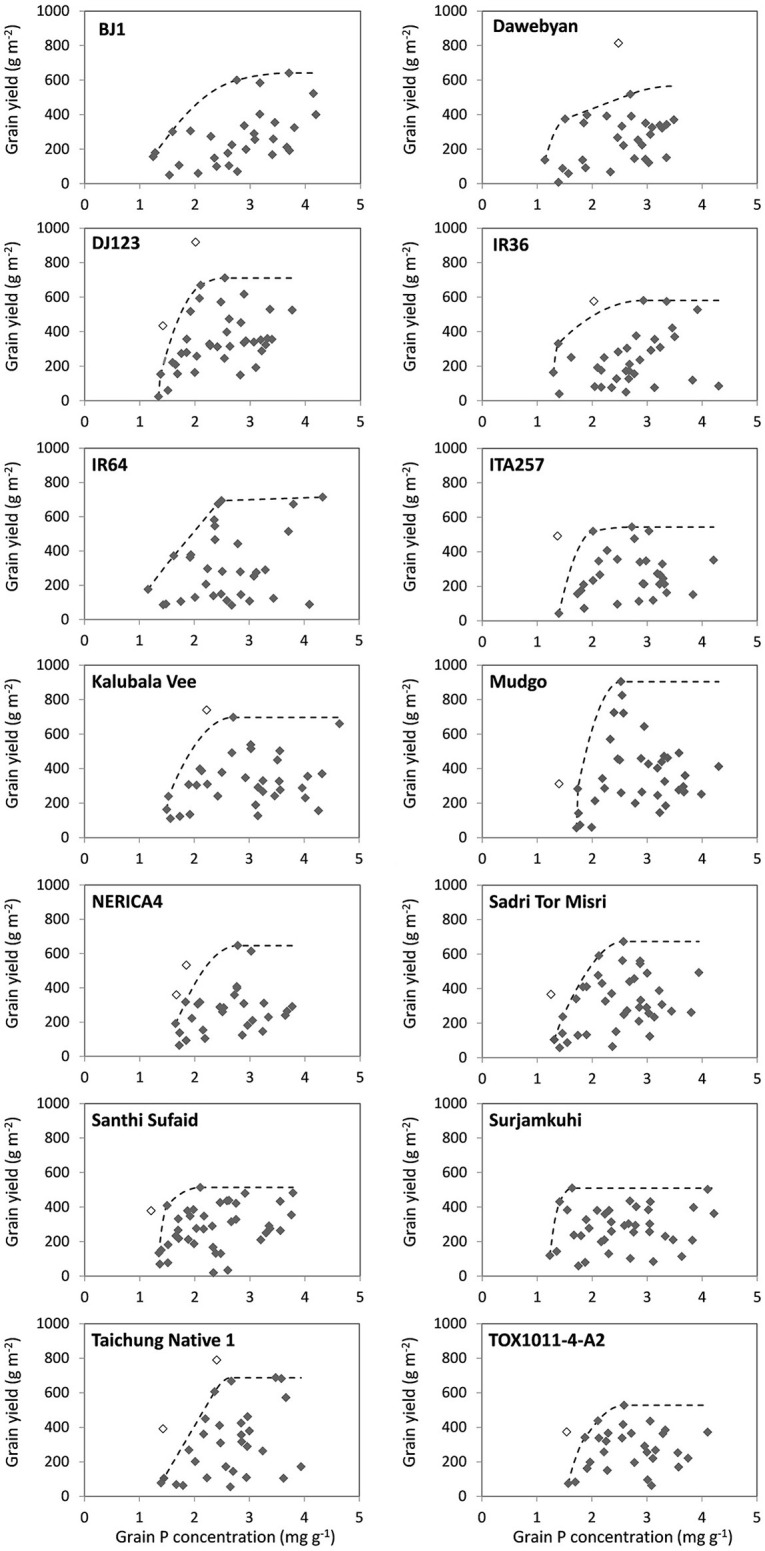
**Grain yield plotted against grain P concentration observed in different environments for 14 rice genotypes, and boundary curves estimating minimum grain P concentrations to reach certain grain yield levels**. Empty dots are outliers not included in the boundary line analysis.

Santhi Sufaid and Surjamkuhi had a relatively low grain yield potential but had a steep slope of the boundary curve below 80% of their maximal grain yield, indicating that these genotypes were able to increase grain yields with only very limited increases in grain P concentrations (Figure 4 and Table 5). Surjamkuhi scored particularly well in terms of minimum grain P concentrations at different grain yield levels, but had relatively high average grain P concentrations, meaning that in many cases it loaded more P than needed (Table 5). Santhi Sufaid did not score particularly well in terms of minimum grain P concentrations, but had low average grain P concentrations at low to medium grain yield levels (Table 5), and was the only genotype for which no grain P concentrations >3 mg g^−1^ were observed at grain yield levels <200 g m^−2^ (Figure 4). The genotypes TOX1011-4-A2 and ITA257 had grain yield potentials similar to those of Santhi Sufaid and Surjamkuhi, but the slope of the lower part of their boundary curves was notably less steep, meaning that these genotypes rapidly accumulated more P in their grains upon an increase in P supply. The genotype Mudgo had the highest grain yield plateau (maximum grain yield), followed by DJ123 and IR64, but these genotypes differed clearly in terms of grain P loading patterns (Figure 4). On the one hand, the slope of the boundary curve of DJ123 was much steeper than that of IR64, indicating that DJ123 efficiently utilized grain P while IR64 rapidly increased grain P loading upon an increase in P supply. On the other hand, Mudgo exhibited a slope of the boundary curve that was comparable to that of DJ123, however, compared to DJ123 and IR64 the lower part of its curve was shifted to the right meaning that in general Mudgo required a higher grain P concentration to reach certain grain yields especially at lower yield levels. Other genotypes exhibited intermediate responses.

**Table 5:** **Minimum grain P concentration at various grain yield levels for 14 rice genotypes and average grain P concentration in different grain yield level intervals, and slope of boundary curves below 80% of maximum grain yield**.

	**Minimum grain P concentration (mg g^−1^) At grain yield level**				**Average grain P concentration (mg g^−1^) At grain yield level**			**Slope of boundary curve below 80% of max grain yield**
	**150 g m^−2^**	**300 g m^−2^**	**500 g m^−2^**	**80% of max grain yield**	**<200 g m^−2^**	**200−400 g m^−2^**	**>400 g m^−2^**	
BJ1	1.2	1.6	2.2	2.2	2.4	3.0	3.4	365
Dawebyan	1.2	1.3	2.5	2.2	2.2	2.7	2.6	240
D1J23	1.4	1.5	1.7	1.8	2.0	2.5	2.5	991
IR36	1.3	1.4	2.1	1.9	2.5	2.6	3.1	378
IR64	1.2	1.4	2.0	2.1	2.4	2.5	3.0	395
ITA257	1.5	1.6	1.9	1.8	2.5	2.9	2.4	1052
Kalubala Vee	1.5	1.6	1.9	2.1	2.5	3.0	3.2	651
Mudgo	1.7	1.7	1.9	2.1	2.3	2.9	3.0	1311
NERICA4	1.7	1.8	2.1	2.1	2.3	2.7	2.6	626
Sadri Tor Misri	1.4	1.6	1.9	2.0	1.9	2.7	2.6	632
Santhi Sufaid	1.4	1.4	1.9	1.5	1.9	2.4	2.7	1868
Surjamkuhi	1.2	1.3	1.6	1.4	2.2	2.7	2.6	1907
Taichung Native 1	1.5	1.8	2.2	2.3	2.6	2.5	2.9	543
TOX1011-4-A2	1.6	1.8	2.3	2.1	2.4	2.8	2.6	681

*Minimum grain P concentration and slopes of boundary curves were determined based on the boundary curves presented in Figure 4*.

Despite the clear differences in grain P loading patterns among genotypes that can be derived from the boundary curves in Figure 4, genotypic differences in minimum and average grain P concentrations were rather small in absolute terms (Table 5). Minimum grain P concentrations associated with grain yields of 150, 300, and 500 g m^−2^ varied between 1.2 and 1.7, 1.3 and 1.8, and 1.7 and 2.2 mg g^−1^ among genotypes respectively. Table 6 shows that reductions in P removal potentially achieved by exploiting genotypic differences would be in the order of magnitude of <0.1, 0.15, and 0.5 g P m^−2^ (equivalent to 1, 1.5 and 5 kg P ha^−1^) at grain yield levels of 150, 300, and 500 g m^−2^ (equivalent to 1500, 3000, and 5000 kg ha^−1^) respectively, and less when commonly grown genotypes such as IR64 or NERICA4 are considered as a reference. P removal from fields was, on average across genotypes, 1.7, 1.8, and 1.4 times larger than P removal at minimum grain P concentrations for grain yield levels of 150, 300, and 500 g m^−2^ respectively (Table 6).

**Table 6:** **Minimum P removal and estimated average P removal at various grain yield levels for 14 rice genotypes**.

	**Minimum P removal (g P m^−2^) At grain yield level**			**Estimated average P removal (g P m^−2^) At grain yield level**		
	**150 g m^−2^**	**300 g m^−2^**	**500 g m^−2^**	**150 g m^−2^**	**300 g m^−2^**	**500 g m^−2^**
BJ1	0.19	0.47	1.08	0.35	0.90	1.70
Dawebyan	0.17	0.40	1.27	0.32	0.81	1.29
D1J23	0.21	0.45	0.87	0.30	0.76	1.25
IR36	0.19	0.41	1.03	0.38	0.79	1.57
IR64	0.17	0.43	0.98	0.36	0.74	1.48
ITA257	0.22	0.48	0.96	0.37	0.86	1.18
Kalubala Vee	0.23	0.48	0.97	0.37	0.89	1.59
Mudgo	0.26	0.52	0.94	0.35	0.87	1.48
NERICA4	0.25	0.54	1.07	0.34	0.82	1.30
Sadri Tor Misri	0.20	0.47	0.95	0.29	0.81	1.28
Santhi Sufaid	0.20	0.42	0.94	0.29	0.72	1.35
Surjamkuhi	0.19	0.39	0.78	0.34	0.80	1.31
Taichung Native 1	0.23	0.54	1.09	0.38	0.75	1.43
TOX1011-4-A2	0.24	0.54	1.16	0.36	0.84	1.29

*Average P removal was estimated based on average P concentrations observed in the grain yield intervals presented in Table 5*.

## Discussion

### Variation in grain P concentration among environments and genotypes

Grain P concentration varied widely among environments and genotypes. Grain yields above 400 g m^−2^ were generally associated with minimum grain P concentrations of around 2 mg g^−1^ (Figure 3). However, at the same grain yield level of 400 g m^−2^, average grain P concentrations up to 4 mg g^−1^ were observed in other environments because of luxury P supply or other constraints limiting a further yield increase. At lower yield levels of about 200 g m^−2^, a similar 2-fold variation in grain P loading was observed. Our observations are comparable with those of Dobermann and Fairhurst (2000), who determined typical grain P concentrations in rice ranging from 1.7 to 2.3 mg g^−1^ under nutrient limitation, 2.4 to 2.8 mg g^−1^ under nutrient optimum, and 2.8 to 4.8 mg g^−1^ under nutrient surplus, and in agreement with the 2-fold range (2–4 kg t^−1^) in P uptake per ton of rice grain yield reported by Dobermann et al. (1998). Especially at higher yield levels, where biomass export from fields is high, such large variation in grain P loading at equal grain yields is expected to have important implications for P removal rates, field P balances and subsequent fertilizer requirements. Indeed, at a yield level of 400 g m^−2^, 1.6 g P m^−2^ (equivalent to 16 kg P ha^−1^) is removed with grains from fields at grain P concentrations of 4 mg g^−1^, compared to only 0.8 g P m^−2^ (equivalent to 8 kg P ha^−1^) at grain P concentrations of 2 mg g^−1^. In high-input, high-yielding systems, matching externally applied P with crop P demand, based on a targeted yield level, therefore appears a logical option to avoiding excess P uptake and excessive P removal from fields. This may involve fine-tuning fertilizer rates or innovative water management to manipulate P availability at different crop development stages. Since yields are known to respond to the most limiting nutrient, a crucial aspect for maximizing nutrient use efficiency and avoid excess accumulation of non-limiting nutrients without a concomitant yield increase is also the balanced use of fertilizers (Janssen, 1998; Dobermann and Fairhurst, 2000). This can be exemplified by the study of Bi et al. (2013) who found that grain P concentrations of rice decreased in response to increasing N rates and concluded this was at least partly due to a dilution effect. At lower grain yield levels, where high grain P loading but low grain yields are observed due to other yield limiting factors, agronomic management needs to focus on overcoming these other stresses to improve the amount of rice harvested per unit of P exported from fields with grains.

Fine-tuning nutrient availability is, however, not a straightforward approach in rice systems where small-scale farming dominates and soil analysis may not be readily available. Exploiting genotypic variation in grain P concentrations to breed P-efficient crop cultivars that minimize P removal from fields may therefore be a more sustainable or practical option (Rose et al., 2010). A major aim of the present study was to investigate whether sufficient variation for grain P concentration exists among the rice genotypes tested to warrant the selection of genotypes as donor varieties in a breeding program. To this end, genotypic variation in grain P concentration was investigated across diverse environments while concomitantly assessing grain yields. The 1.3–2.7-fold variation in grain P concentration within environments observed in this study is in agreement with earlier field studies that have evaluated genotypic variation in grain P concentrations in one environment for various cereals including rice (Rose et al., 2010), sorghum (*Sorghum bicolor*) (Leiser et al., 2014) and maize (*Zea mays*) (Wardyn and Russell, 1998). However, the roughly 2-fold variation in grain P concentration observed within environments does not imply that grain P concentration could be reduced by 50% through breeding. Within the set of genotypes tested, genotypes with exceptionally high grain P concentrations were also included, and furthermore, a considerable part of the genotypic variation in grain P concentration within environments was related to differences in grain yield. The most promising genotype appeared to be Santhi Sufaid as it exhibited considerably low grain P concentrations in all types of environments irrespective of its grain yield level, while the genotype DJ123 was the only genotype that had lower than average grain P concentrations in all environment clusters combined with medium to high yield. The same genotypes were found in previous studies to exhibit high PUE at the vegetative stage (Saito et al., 2015; Rose et al., 2016). The boundary line analysis showed that by using Santhi Sufaid as a donor in breeding for low grain P concentration, a reduction of 5 to maximally 20% in grain P concentration at equal yield levels can be achieved in popular rice genotypes such as IR64 and NERICA4.

### Relationship between grain P concentration and grain yield and PUE

Different patterns with regard to the interaction between grain P concentration and grain yield could be distinguished. A first pattern involved genotypes with low grain P concentrations and medium to high grain yields, and can be exemplified by the performance of Kasalath in P-limited environments, which seemed to have a superior P utilization efficiency. A second pattern involving high grain P concentrations combined with medium to high grain yields was observed for the genotypes Kalubala Vee and Mudgo. For these genotypes, high grain P concentrations appeared to be the result of an outstanding P uptake capacity. The high P uptake capacity of Mudgo is in agreement with the results of Saito et al. (2015), where Mudgo had the highest P uptake among 7 genotypes, measured at the vegetative stage. At maturity, Mudgo was found to have 10–20% higher grain P concentrations and 20–50% higher straw P concentrations than Santhi Sufaid while yield levels were similar (Vandamme et al., 2016). For Kalubala Vee, the excessive P loading was at least partly the result of enhanced remobilization of P from the straw to grains during grain filling, as straw P concentrations for this genotype were exceptionally low across environments. A third pattern involved genotypes with low grain P concentrations combined with low grain yields, and this was observed for Tondok which nevertheless also had high straw P concentrations, indicating that for this genotype a major problem occurred with mobilizing resources to the panicles for grain production.

Reduced grain P concentration may either be the result of high PUE (biomass produced per unit of P uptake) in general (low P concentrations in all parts of the plant) or of a reduced translocation of P to the grains at equal grain yield which leads to a lower P harvest index (PHI) (Rose et al., 2013; Vandamme et al., 2015). In the first case, P concentration is expected to be lower than average in both grains and straw, and this was observed for a number of genotypes such as Kasalath and DJ123, and Santhi Sufaid at low to medium grain yield levels. This is not surprising for DJ123 and Santhi Sufaid, which have high PUE during vegetative growth (Saito et al., 2015; Rose et al., 2016). In the second case (lower PHI at equal grain yield), low grain P concentrations combined with higher than average straw P concentrations are expected. However, at maturity, straw P concentrations are affected by P mobilization to the grains which is in turn determined by a combination of all grain yield-determining factors contributing to the harvest index. To determine whether low grain P concentrations are the result of general PUE or lowered PHI at equal grain yield, data from grain P concentrations at maturity need to be compared with data from vegetative phase screening for PUE. In recent studies by Wissuwa et al. (2015) and Rose et al. (2016), where PUE at the vegetative stage was compared among genotypes at equal total plant P content, Santhi Sufaid and DJ123 exhibited high PUE, indicating that the low grain P concentrations observed in these genotypes were indeed related to whole plant PUE.

### Improvements of field P balances given current levels of genotypic differences in grain P concentrations

The boundary line analysis showed that at low grain yield levels of around 150 g m^−2^, potential improvements in field P balances that can be achieved by exploiting genotypic variation in minimum grain P concentration observed within the limits of this study are less than 0.1 g P m^−2^ (equivalent to 1 kg P ha^−1^), which is a rather small amount of P especially in the short term. Based on this genotypic variation, breeding for low grain P concentration using a conventional approach does not seem to be a viable option for reducing P mining in low-yielding, low-input systems. At higher yield levels (300–500 g m^−2^), boundary curve analysis showed that breeding for low grain P concentration can improve field P balances by 0.15 to maximally 0.5 g P m^−2^ (1.5 to maximally 5.0 kg P ha^−1^). This may seem a modest amount of P in absolute terms, particularly compared to generally recommended P application rates of 15–60 kg P_2_O_5_ ha^−1^ (= 6.5–26 kg P ha^−1^) for rice (Fairhurst et al., 2007). However, in relative terms it constitutes a reduction of 20–40% in P removal with grains, and this would hence considerably reduce the maintenance fertilizer need or the amount of P mined in low input systems.

Reductions in P removal larger than estimated above are possible since boundary conditions (Shatar and McBratney, 2004) represent ideal cases when the utilization of P is optimized within the plant (any reduction in P uptake would reduce yield). These conditions rarely exist and data in Figure 4 show that grain P concentrations at yield levels of 400–500 g m^−2^ can be 2-fold higher than boundary levels within each genotype. A highly P efficient genotype like DJ123 can achieve that yield level at a grain P concentration of 1.5–1.7 mg g^−1^ under optimized conditions. One could use this P concentration in setting a target concentration in breeding efforts to be achieved not only at boundary conditions but across environments, thus effectively avoiding any excess loading of P into grains. This would reduce P offtake from fields and therefore reduce maintenance fertilizer requirements beyond levels discussed above. While excess loading of P is of no agronomic concern in high-input systems (because mining is not a concern), it should be limited to reduce the environmental impact associated with the consumption and poor utilization of P (Withers et al., 2001) and the negative effects on human and animal health related to phytate-rich grains (Raboy, 2009). Nevertheless, as desirable as it should be, capping P loading at some low P concentration around 1.5 mg g^−1^ does not appear feasible using conventional breeding as none of the genotypes studied had the ability to fully restrict luxury P loading into grains. Santhi Sufaid had a lower tendency to load excess P under conditions of additional stresses compared to other genotypes, but on average still loaded around 50% P more than minimum levels needed to reach certain yield levels.

### Toward developing rice varieties with reduced grain P loading

Consistent genotypic variation in grain P concentration of rice was observed across a wide range of rice growing environments, and Santhi Sufaid and DJ123 were identified as potential donors for breeding for low grain P concentrations. Improvements in P balances that could be achieved by exploiting this genotypic variation are in the range of less than 1 kg P ha^−1^ in low yielding P deficient environments, and 1.5–5 kg P ha^−1^ in higher yielding systems. The magnitude of that potential improvement is likely too small to justify breeding activities specifically targeting this trait, particularly since the current lack of selectable markers and the high degree of environmental variation constitute technical barriers for the successful implementation of a traditional breeding program for reduced grain P concentrations. A larger portion of the rice gene pool may have to be screened, ideally in a genome wide association approach, to identify donors and associated markers. In addition alternative options should be explored that may include screens of mutant populations (Raboy, 2009) and identification of candidate genes involved in the regulation of P loading into grains for potential genetic manipulation (Wang et al., 2015; Jeong et al., 2016).

The present study demonstrated that relatively high yield levels (>4 t ha^−1^) were achieved at grain P concentrations around 1.5–1.7 mg g^−1^, and Pariasca-Tanaka et al. (2015) showed that grain P concentrations as low as 1 mg g^−1^ did not affect seedling vigor in some rice genotypes. Currently it is not known at what level a “safe” lower limit for grain P concentration that does not affect subsequent crop productivity would be, and additional research is needed to clarify this point. However, conceptually it is evident that maximum benefits in terms of reducing P mining, maintenance P requirements and environmental P fluxes would be achieved by efforts to cap grain P concentrations at such a lower limit by avoiding excess P loading to grains. Large (2-fold) across-environment variation in grain P concentrations at equal grain yield levels indicated that such excess P uptake is common and likely due to other yield-limiting factors leading to less than optimal P utilization efficiency. Any effort to lower P removal by grains through genetic improvement should therefore be combined with improved crop management to overcome other yield-limiting factors and maximize utilization efficiency of P in grains and ultimately in cropping systems.

## Author contributions

KS coordinated data collection in Africa and MW in Asia. KD, MF, KS, RV, DJ, ZS, LS, DS, YK, and TR conducted field trials and analyzed samples. EV, KS, and ID compiled and analyzed the data. EV wrote the manuscript with contributions from all co-authors. All co-authors read and approved the final manuscript.

### Conflict of interest statement

The authors declare that the research was conducted in the absence of any commercial or financial relationships that could be construed as a potential conflict of interest.

## Supplementary material

Supplementary Table 1 **Information on the materials and methods used in each of the multi-location trials including location, rice growing environment, year, season, soil characteristics, fertilizer rates, experimental design, plot size, hill density, number of replicates and total number of genotypes for 41 environments**.

Supplementary Datasheet 1 **Genotype × environment least square means for grain yield, straw biomass, grain P concentration and straw P concentration**.
